# Muscle Activity Adaptations to Spinal Tissue Creep in the Presence of Muscle Fatigue

**DOI:** 10.1371/journal.pone.0149076

**Published:** 2016-02-11

**Authors:** Jacques Abboud, François Nougarou, Martin Descarreaux

**Affiliations:** 1 Département d’Anatomie, Université du Québec à Trois-Rivières, Québec, Canada; 2 Département de Génie Électrique, Université du Québec à Trois-Rivières, Québec, Canada; 3 Département des Sciences de l’Activité Physique, Université du Québec à Trois-Rivières, Québec, Canada; University of Palermo, ITALY

## Abstract

**Aim:**

The aim of this study was to identify adaptations in muscle activity distribution to spinal tissue creep in presence of muscle fatigue.

**Methods:**

Twenty-three healthy participants performed a fatigue task before and after 30 minutes of passive spinal tissue deformation in flexion. Right and left erector spinae activity was recorded using large-arrays surface electromyography (EMG). To characterize muscle activity distribution, dispersion was used. During the fatigue task, EMG amplitude root mean square (RMS), median frequency and dispersion in x- and y-axis were compared before and after spinal creep.

**Results:**

Important fatigue-related changes in EMG median frequency were observed during muscle fatigue. Median frequency values showed a significant main creep effect, with lower median frequency values on the left side under the creep condition (*p*≤0.0001). A significant main creep effect on RMS values was also observed as RMS values were higher after creep deformation on the right side (*p* = 0.014); a similar tendency, although not significant, was observed on the left side (*p =* 0.06). A significant creep effects for x-axis dispersion values was observed, with higher dispersion values following the deformation protocol on the left side (*p*≤0.001). Regarding y-axis dispersion values, a significant creep x fatigue interaction effect was observed on the left side (*p* = 0.016); a similar tendency, although not significant, was observed on the right side (*p* = 0.08).

**Conclusion:**

Combined muscle fatigue and creep deformation of spinal tissues led to changes in muscle activity amplitude, frequency domain and distribution.

## Introduction

Spine stability is often described as a complex mechanism involving three essential components: spinal muscles, passive spinal tissues and neuromuscular control [1]. Under normal conditions, these subsystems are highly coordinated and optimized to provide adequate stability of the spine. However, in the absence of muscles to provide spinal stability, compressive loads as low as 100 N can lead to buckling of the entire lumbar spine [2]. Conversely, when spinal muscles are activated, individuals can withhold spinal loads of 4000 N without reporting any pain or undesirable effects [3]. Moreover, it has been hypothesized that changes in muscle recruitment patterns, such as muscle co-contraction, act as compensation for spinal instability resulting from passive elements laxity or reduced neuromuscular control [1, 4].

Such adaptations in muscle recruitement patterns can also be observed with the use of large-arrays surface electromyography (EMG) under the influence of muscle fatigue [5]. Indeed, a migration of muscle activity has been described during a low back muscle fatigue task [5, 6]. Moreover, large-arrays surface EMG has been shown to be a relevant tool in the identification of distinctive muscle recruitment strategies between sympatomatic vs asymptomatic individuals in different low back regions through different motor tasks [7, 8]. Zwarts showed that large-arrays surface EMG offers unique spatial information to our knowledge regarding the distribution of muscle activity, such as motor unit activation. [9].

On the other hand, spinal instability associated with a deformation of passive spinal tissues, as it occurs in repetitive exposure to prolonged deep trunk flexion, has been associated with the development or occurrence of low back pain (LBP) and disorders [10–12]. This association could partly be explained by the combination of gradually increasing creep in the viscoelastic tissues and decreases in reflexive muscular activation that leave the spine with diminished protection from instability [13]. Experimentally induced low back muscle creep has been used in several studies to further our understanding of the passive and active structure contribution to spinal stability [14–21]. In these studies, active (dynamic) or passive (static) flexion-extensions of the trunk are the most commonly used protocols to induce spinal creep. These sustained or repeated movements usually lead to an increase in the trunk flexion range of motion [15–18, 22]. Indeed, the creep in the spine ligaments is thought to increase the intervertebral joints laxity, allowing increased relative motion.

The laxity developed in the spine from the creep in the viscoelastic tissues of ligaments, discs and joint capsules is relatively small, and can easily be compensated by moderate adjustments in the co-contraction levels of agonist and antagonist muscles [23]. However, the effects of active or passive prolonged deep flexions of the trunk on low back muscle activity are not well understood. Indeed, no change has been observed in the timing of muscle activation onset for the lower erector spinae muscles following a static passive lumbar flexion period of 10 [19] or 15 minutes [14], whereas onset latency of the same muscles increased after one hour of static passive lumbar flexion [20]. Olson et al. showed that no difference exists regarding onset latency of the lower erector spinae muscles following either active or passive trunk flexion-extension repetitions [21]. Furthermore, human in vivo studies indicate that a prolonged trunk flexion results in a higher paraspinal muscle reflex gain [14], while repeatedly applied short-duration creep reduces spinal reflex responses [15]. Decreased protective muscular reflex was shown to be the direct manifestation of mechanoreceptor desensitization caused by laxity in the viscoelastic tissues of the spine [23]. Lastly, the spinal stabilizing system acts by altering muscle activation patterns via the nervous system in response to the ligamentous tissue mechanoreceptor afferent signals. Spinal muscular activity is then generated in order to compensate the decreased contribution of viscoelastic tissues by implementing alternative recruitment strategies such as the co-contraction of trunk muscles [4, 24].

Under the influence of back muscle fatigue, a similar reorganization of motor strategies is implemented to perform the fatigue task. Indeed, many studies have shown adaptations in recruitment patterns during muscle fatigue [6, 8, 25]. Moreover, low back muscle fatigue seems to be associated with changes in motor reflex activity following unexpected postural perturbation [26–28]. Additionnally, creep deformation has been shown to alter passive structures, which are known to play a crucial role in lower back spinal stability [1].

Arjmand and Shirazi suggested that static flexion of the trunk, which induces creep deformation of the passive structures, may be a significant risk factor for low back disorders or development of muscle fatigue [29] and can possibly increase the demand on other trunk stabilizing structures.

The aim of this study was to describe the effect of spinal tissue creep on muscle activity distribution in presence of muscle fatigue. Based on previous studies showing that increases in EMG amplitude signals are observed under the influence of low back creep deformation, it was hypothesized that back muscle recruitment strategies, which are believed to play an important role in redistributing stabilization efforts, will be modified following a soft tissue creep in the spine.

## Materials and Methods

### Participants

Twenty-three healthy adult participants without history of LBP were recruited. This group was composed of 12 women and 11 men (mean (SD): age = 26.7 years (5.1); height = 1.70 m (0.1); weight = 67.7 kg (14.2); BMI = 23.2 kg/m^2^ (3.9)). Exclusion criteria were: any history of acute/chronic thoracic or low back pain in the past 6 months, ankylosing spondylitis, trunk neuromuscular disease, inflammatory arthritis, scoliosis (≥ 15°), and previous spinal surgery. The project received approval from the University’s ethics committee for research with humans (Comité d'éthique de la recherche avec des êtres humains) and all participants gave their written informed consent prior to their participation in the study.

### Experimental protocol

Each subject participated in one initial experimentation during which they performed a maximal voluntary isometric trunk extension contraction (MVC), range of motion (ROM) assessment and a 1-minute fatigue task before being submitted to the 30-minute passive tissue deformation condition. After creep deformation was obtained, the ROM assessment and the fatigue task were conducted again. All together, the two ROM tests were repeated 3 times each. First, the trunk angle was measured by placing the digital inclinometer (Dualer IQ Pro™ Digital Inclinometer, JTECH Medical; USA) on the L3 vertebra. The participants stood upright and then tilted the trunk forward as much as possible, without bending the knees. The ROM was also measured by asking the participant to sit on the floor and completely rest the soles of their feet against the standard Flex-Tester (Baseline Sit n’ Reach Box, Fabrication Enterprises Inc., USA). With their arms and fingers in full extension in front of them, participants were asked to push the metal plate the farthest they could, without bending the knees so that the trunk leaned forward as much as possible. The flexion position was maintained for 2 seconds before participants were allowed to sit upright again.

The MVC protocol was performed prior to the fatigue protocol. Participants were asked to lay in a prone position on a 45° Roman chair, with the iliac crests aligned with the chair cushion edge. A belt fixed to the ground and installed over participants shoulders resisted the force. The fatigue protocol consisted of a modified version of the Sorensen endurance test [30], executed in the same position as the MVC protocol. In order to quickly induce muscular fatigue, participants had to lift a 12.5-kilogram weight plate during the task. The plate was held as close as possible to the chest by the participants. The participants’ trunk was maintained unsupported in a horizontal position relative to the ground for one minute. Immediately after the fatigue protocol, participants were asked to perform the deformation protocol. Perceived effort scale (6–20) [31], measuring the intensity of the fatigue task, was rated by each participant at the end of the fatigue test.

**Fig 1:**
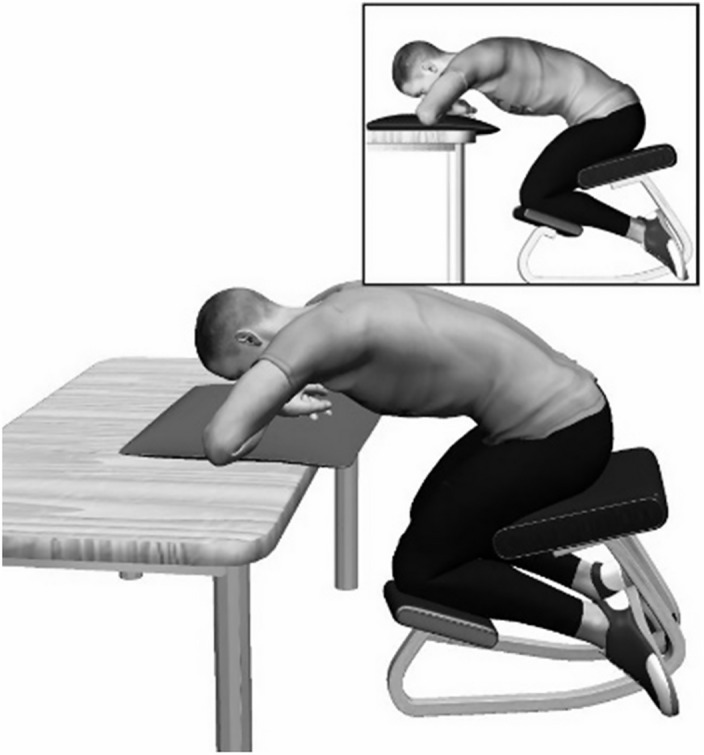
Illustration of the low back creep deformation protocol.

The low back creep deformation protocol started with the participants sitting on a bench and then bending forward, so that their trunk was supported by a table (Fig 1). Participants’ trunks were flexed by approximately 75% of their range of full trunk flexion. Their legs were also flexed by 90 degrees to limit the occurrence of harmstring muscles stretching. They maintained this position for 30 minutes. Immediately after, the ROM was measured again by the 2 previously mentioned tests, and the fatigue tasks were performed a second time afterwards. Lumbar muscles’ activation (EMG) was obtained only during the fatigue task and consequently assessed before and after the deformation protocol.

### Data Acquisition

**Fig 2:**
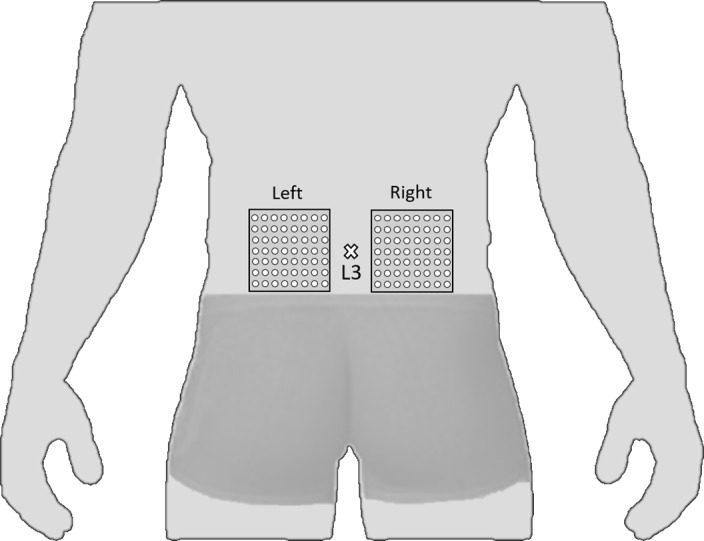
Representation of two 64-electrode matrices used in the recording of erector spinae muscle activity (model ELSCH064; LISiN-OT Bioelettronica, Torino, Italy).

Right and left erector spinae’ activity was recorded using two large-arrays surface EMG matrices (model ELSCH064; LISiN-OT Bioelettronica; Torino, Italy). The array grid consisted of 64 electrodes placed in an 8x8 matrix (10 mm inter-electrode distance). The center of each grid was located at L3 level (Fig 2), and one ground electrode was placed on the left ulnar process. Skin impedance was reduced by shaving body hair, gently exfoliating the skin with fine-grade sandpaper (Red DotTrace Prep, 3 M; St. Paul, MN, USA) and wiping the skin with alcohol swabs. The bipolar EMG signals were amplified (64-channel surface EMG amplifier, SEA 64, LISiN-OT Bioelettronica; Torino, Italy; –3 dB bandwidth 10–500 Hz) by a factor of 2000, sampled at 2048 Hz and converted to digital form by a 12 bit A/D converter. The data were collected using the OT Bioelettronica custom software and processed by Matlab (MathWorks; Natick, MA, USA).

### Data Analysis

The ROM measured in a standing position using an inclinometer was assessed following the American Medical Association recommendations [32]. To establish the initial position, the individuals were standing with their knees extended and their weight balanced on both feet (spine was in a neutral position). The position of each participant was verified by the same experimenter to limit measurement errors. The Sit n’ Reach test was performed using the procedures outlined in the American College of Sports Medecine (ACSM) manual [33]. For both ROM tests, the highest value from the 3 trials was considered for all analyses.

Each bipolar EMG signal obtained from both matrices was digitally band-pass filtered in the frequency bandwidth 20-450Hz (2^nd^ order Butterworth filter). Notch filters were also applied to eliminate the 60Hz power line interference and its harmonics. As described in a previous study, dispersion was obtained by calculating the center of gravity dispersion during the Sorensen test of a given subject [8]. In short, myoelectric signals from each electrode were normalized with the baseline EMG signal obtained from MVC trials. Each electrode-filtered signal was then divided in *L* windows of 0.5s for which an individual root mean square (RMS) value was computed. For each window, the mean of all electrodes RMS was calculated and correspond the centroid position. To characterize muscle activity distribution, the dispersion variable in x- and y-axis, representing the muscle activity range of displacement (centroid), was extracted from the bipolar EMG signals. The x-axis corresponds to the mediolateral direction, while the y-axis corresponds to the cephalic-caudal direction. In order to confirm the presence of muscle fatigue in both conditions (no creep and creep), the mean normalized slope of the median frequency (MDF) (mean of the 64 electrodes of each matrix) was calculated using the same methods as the one used for RMS. The Fourier transform function from Matlab software (MathWorks; Natick, MA, USA) was used to calculate the MDF values. The MDF was defined as the frequency that divided the spectrum into two equal areas. Each signals was then divided in 0.5s windows without overlap for which an MDF value was computed [34]. More specifically, MDF values were obtained through each electrode signal. The slope of MDF was then calculated for each electrode. Finally, a value representing the mean slope of MDF was obtained.

### Statistical analyses

Normality of distribution for every dependent variable was assessed using the Kolmogorov–Smirnov test, in addition to visual inspection of the data. The *t-test* for dependent samples was used to compare rates of perceived effort at the end of the fatigue test before and after the deformation protocol. Ranges of motion measured by the digital inclinometer and the “Sit n’ Reach Box” were also compared before and after the deformation protocol using the *t-test* for dependent samples. The *t-test* for dependent samples was also used to compare normalized MDF slopes before and after the deformation protocol. EMG muscle variables (mean MDF, mean RMS, dispersion in x- and y-axis) data were compared between the two conditions (no creep and creep) using a two-way repeated-measure analysis of variance (ANOVA) to assess the main effects of creep and fatigue (early fatigue: 10 first seconds and late fatigue: 10 last seconds of the Sorensen test). When necessary, the Tukey post hoc test was performed as the post hoc analyses for pair-wise comparisons. For all statistical analyses, p<0.05 was considered to be statistically significant.

## Results

From the 23 original participants, three were excluded from EMG analyses due to high levels of EMG noise in recordings.

### Impacts of muscle fatigue

Important fatigue-related changes in sEMG time-frequency were observed during the fatigue protocol. Dependent t-tests revealed no significant difference between the before-deformation condition (on the left side: mean = –0.30; SD = 0.09 and on the right side: mean = –0.29; SD = 0.12) and after-deformation condition (on the left side: mean = –0.29; SD = 0.10 and on the right side: mean = –0.27; SD = 0.19) regarding the normalized MDF slopes (*p≥0*.*05*). There was also no difference regarding the rated perceived effort (before deformation, mean = 13.2/20; SD = 2.9 and after deformation, mean = 13.6/20; SD = 3.2) (*p>0*.*05*).

### Creep deformation effects

#### Range of motion

Regarding the ROM comparison before and after creep deformation, results showed a significant increase in ROM after the deformation protocol measured by the Sit n’ Reach Box test (*p* = 0.02). However, no difference was found regarding ROM measured by the inclinometer (*p* = 0.42).

#### MDF

**Fig 3:**
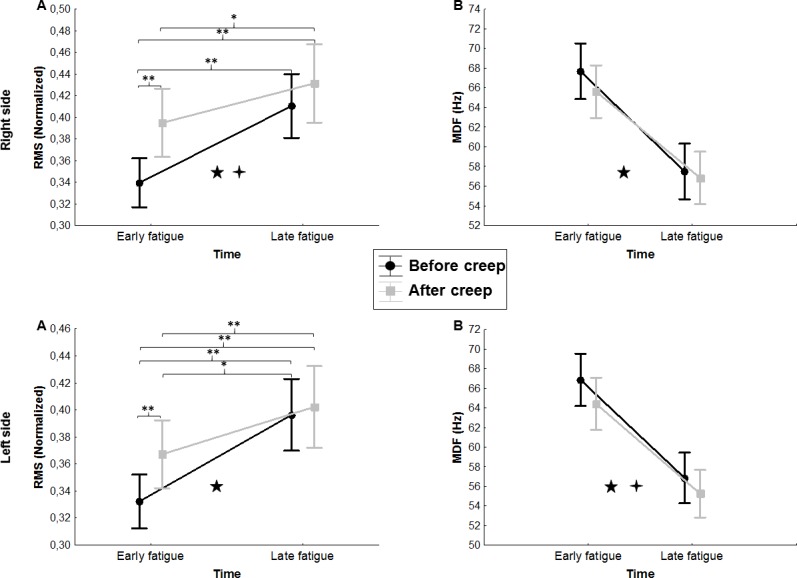
**Mean RMS (A) and MDF (B) values over time on the right and left sides** (RMS: Root Mean Square; MDF: Median Frequency). Error bars indicate standard deviations. ★ represents a main effect of fatigue. ✦ represents a main effect of creep. Post hoc results are illustrated by * = *p* ˂ 0.01 and ** = *p* ˂ 0.001.

The ANOVA revealed main significant fatigue effects on MDF for both conditions. As expected, MDF was lower at the end of the fatigue task in comparison to its beginning on the right side [*F*(1,19) = 80.73, *p* ≤ 0.0001] and the left side [*F*(1,19) = 178.51, *p* ≤ 0.0001]. Moreover, results of MDF values showed a significant main creep effect, with lower MDF values on the left side [*F*(1,19) = 38.73, *p* ≤ 0.0001] under the creep condition, but no difference was observed on the right side [*F*(1,19) = 2.37, *p* = 0.14] (Fig 3B).

#### RMS

The ANOVA revealed a significant main creep effect on mean RMS values, as RMS was higher after creep deformation on the right side [*F*(1,19) = 7.31, *p* = 0.014] and a similar tendendy, although not significant, was observed on the left side [*F*(1,19) = 3.92, *p =* 0.06]. The ANOVA also revealed a significant main fatigue effect on mean RMS values. RMS values were higher at the end of the fatigue task on the right side [*F*(1,19) = 25.70, *p* ≤ 0.0001] and the left side [*F*(1,19) = 32.20, *p* ≤ 0.0001]. Finally, the analyses also revealed a significant interaction effect (creep x fatigue) on the right [*F*(1,19) = 7.26, *p* = 0.014] and left side [*F*(1,19) = 8.93, *p* = 0.008]. As illustrated in Fig 3A, post hoc analyses revealed significant differences during the first 10 seconds, with higher RMS values under the creep condition.

#### X-axis dispersion

**Fig 4:**
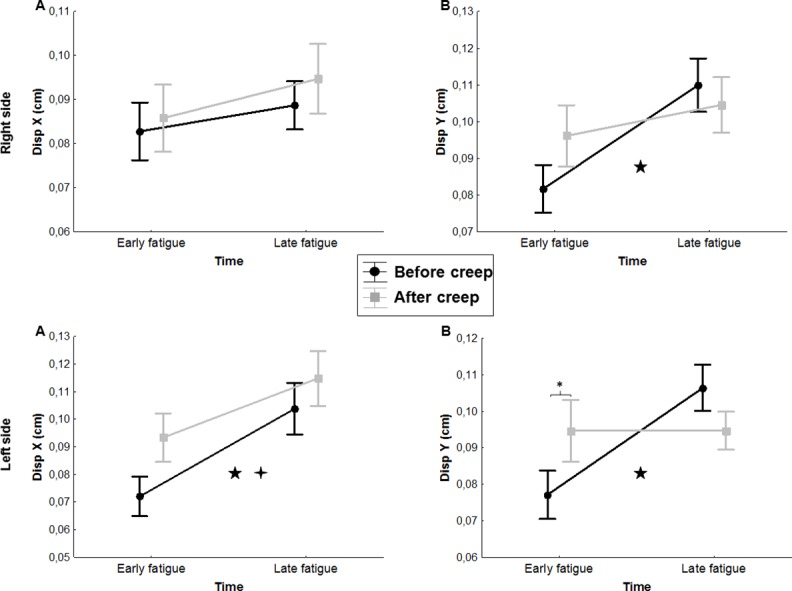
**Mean dispersion values in x-axis (A) and y-axis (B) values over time on the right and left sides** (Disp X: Dispersion in x-axis; Disp Y: Dispersion in y-axis). Error bars indicate standard deviations. ★ represents a main effect of fatigue. ✦ represents a main effect of creep. Post hoc results are illustrated by * = *p* ˂ 0.01.

**Fig 5:**
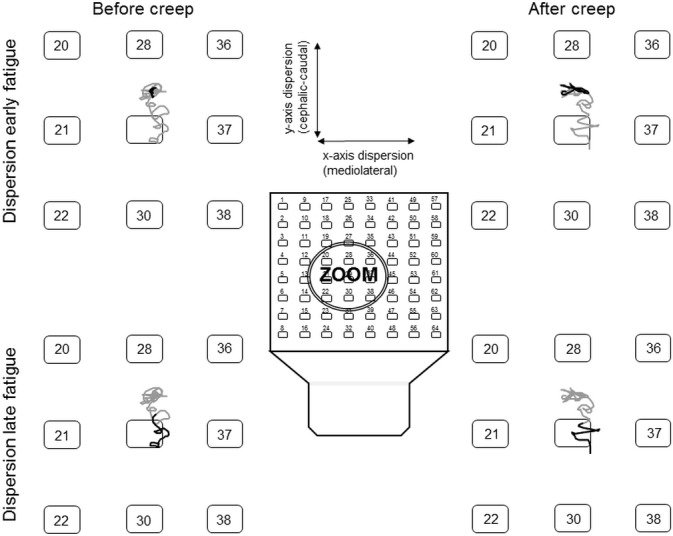
Typical representation of dispersion data from a participant on the right erector spinae. The large-array EMG was enlarged to better observed dispersion data. Bold black lines illustrate the migration of the centroid during the early (upper part of the figure) and late (lower part of the figure) muscle fatigue. Note the shift in the distribution of EMG amplitude toward the caudal region of the lumbar erector spinae (grey lines).

The ANOVA revealed main significant creep effects for x-axis dispersion values, with higher dispersion values following the deformation protocol on the left side[*F*(1,19) = 5.92, *p* ≤ 0.001], but not on the right side [*F*(1,19) = 0.86, *p =* 0.36]. Moreover, the ANOVA showed a significant main fatigue effect on x-axis dispersion values where dispersion was significantly higher by the end of the fatigue protocol on the left side [*F*(1,19) = 17.0, *p* ≤ 0.001], but not on the right side [*F*(1,19) = 1.43, *p* = 0.25] (Fig 4A). Reprensatative data of the dispersion are presented in Fig 5.

#### Y-axis dispersion

The ANOVA revealed main significant fatigue effects on y-axis dispersion values where dispersion was significantly higher by the end of the fatigue protocol on the right side [*F*(1,19) = 7.49, *p* = 0.013] and on the left side [*F*(1,19) = 5.77, *p* = 0.027]. The analyses also showed a significant creep x fatigue interaction effect on the left side [*F*(1,19) = 6.95, *p* = 0.016]; a similar tendency, although not significant, was observed on the right side [*F*(1,19) = 3.44, *p* = 0.08] (Fig 3B). As illustrated in Fig 4B, post hoc analyses revealed higher dispersion values on the y-axis under the creep condition during the first 10 seconds of the fatigue task. Reprensatative data of the dispersion are presented in Fig 5.

## Discussion

The study’s main objective was to identify muscle activity adaptations to spinal tissue creep in the presence of muscle fatigue. The results showed that prolonged flexion of the trunk led to adaptations in muscle activity distribution. Indeed, higher values of amplitude from lumbar erector spinae myoelectric signals, as well as higher dispersion values, were observed in the presence of spinal tissue creep.

To confirm the presence of spinal tissue creep, ROM values were calculated before and after the deformation protocol. Although limited, an increase in ROM was observed following spinal tissue creep. Overall, the increase in full flexion angle might result from the combined viscoelastic elongation of hamstring and erector spinae muscles. Despite the overall tendency observed, the two ROM tests yielded different results. Such disparity could be explained by the differences between the tasks and instruments. The standing position, as previously described, is associated to a co-contraction phenomenon of posterior and anterior trunk muscles which could possibly explained a decreased ROM in full flexion of the trunk. Indeed, an increase in trunk muscle activity has been shown in standing posture when compared to sitting posture [35]. It is also possible that the activation of the anterior muscles (rectus abdominis) contributes to an increased ability of the trunk to travel through a greater range of trunk flexion. Using an inclinometer, instead of a dual inclinometer, provides a global evaluation of the lower limb and back antigravity muscles, whereas a dual inclinometer should be considered to assess the specific lumbar spine changes following creep deformation [36].

In the present study, participants were asked to perform a trunk extensor muscle fatigue task for one minute. The presence of an acute lower back muscle fatigue phenomenon was indicated by a marked decrease in the mean MDF slope, which is considered a reliable indicator of muscle fatigue [37], as well as a score of the rated perceived effort scale categorized between “somewhat hard” and “hard”. Moreover, similar negative slopes were found before and after the deformation protocol, and participants rated similar perception of effort scores at the end of the fatigue task. These results, as a whole, indicate that participants seemed to be in a similar state of fatigue in both conditions (no creep and creep). On the other hand, differences appeared when the mean MDF was considered. Indeed, lower values of mean MDF were observed in the presence of spinal tissue creep. During early fatigue, MDF values are determined by muscle fiber type distribution [38]. The frequency shift towards lower MDF, in the presence of muscle fatigue, has been associated to changes in motor unit recruitement and synchronisation as well as changes in conduction velocity and muscle fiber types [39, 40]. These results are in accordance with those of Shin and al., who observed a reduction in median power frequency during submaximal extension contraction of 15 and 30% of participants’ 5-seconds maximal voluntary contractions, following a static trunk flexion [41]. These observations taken together, could suggest that following spinal tissue creep, fatigue-related phenomenoms are facilitated. It can be hypothesized that prolonged trunk flexion leads to passive tissue deformartion which may increase trunk muscle contribution to spinal stabilization mechanisms during fatigue. Another study conducted by Howarth et al. found a reduction in median power frequency after repetitive active spine flexions; however, this difference was not statistically significant [18]. In this study, the median power frequency was assessed during a Sorensen test lasting 5 seconds, whereas recordings lasted one minute in the present study. Methodological considerations might therefore explain the differences observed between the results of both studies.

With regard to muscle activation amplitude (RMS values), higher values were observed immediately following the deformation protocol. It is important to note that the second fatigue task was conducted 30 minutes after the first one, and it is unlikely that the increase in RMS was associated to a residual effect of the first fatigue task. Indeed, Larivière et al. showed that a rest period of 10 to 15 minutes following a back muscle fatigue task is enough to allow complete back muscle recovery [42].

RMS values were similar in both conditions at the end of the fatigue protocol, with a tendency towards higher values after the deformation protocol. In the presence of muscle fatigue, an increase of EMG amplitude signals was also observed in the previous study 5 or 10 minutes after performing lifting trials in static lumbar full flexion [16, 41]. Indeed, the increased activity of extensor muscles suggests that increased muscle activation is required to generate more active forces to compensate for the loss of contribution of passive tissues to spinal stability [43]. Moreover, Toosizadeh et al. showed that trunk muscle activity measured by RMS increased by 5% following creep deformation induced by repetitive lifting tasks [44]. Given that the moment arms of paraspinal muscles are relatively small [45], only small increases in muscle forces are needed to increase spinal loads and compensate for reduced spinal stability.

It is well known that neurophysiological perturbations, such as muscle fatigue or musculoskeletal pain, are associated with altered muscles recruitment distribution [7, 8, 46]. The present study is the first one investigating muscle activity distribution in the presence of spinal tissue creep. Dispersion of muscle activity, representing the muscle activity range of displacement, was used to identify recruitment strategies adaptations during the fatigue task before and after the deformation protocol. An increase in dispersion values was observed throughout the fatigue task, which is in accordance with previous studies [5, 8]. Indeed, a caudal and lateral shift of the muscle activity was observed in the current study. This findings support other studies that specifically reported a caudal shift of back muscle activity centroid during muscle fatigue in asymptomatic participants [5, 6]. This observation suggests variations in the distribution of muscle fiber types throughout segments of lumbar erector spinae. Indeed, a larger decrease in EMG power spectral frequency, as well as a simultaneous increase in EMG amplitude have also been showed to the lower part of lumbar muscles in comparison to upper part under the influence of muscle fatigue [47, 48]. Moreover, higher dispersion values were observed at the beginning of the fatigue task under the spinal tissue creep condition. As discussed earlier, adaptations in recruitment strategies, such as co-contraction of trunk muscles, have been described as potential strategies to compensate the decreased contribution of viscoelastic tissues [4, 24]. The present study shows that muscle activity adaptations could also occur within the same muscle through an increase in muscle activity distribution in response to spinal tissue creep. It can be hypothesized that long-lasting creep deformation will increase spinal passive tissue mechanoreceptors threshold, leading to increased muscle activity during static lumbar extension. Ligaments contain mechanoreceptors acting as transducers, sending postural information to the central nervous system. Therefore, following a spinal tissue creep and under postural instability, the central nervous system replies via an appropriate and coordinated feedback muscular action [1, 49]. This suggests that spinal tissue creep yields neuromuscular adaptations (redistribution of muscle activity) similar to those observed under muscle fatigue or muscular pain conditions. Furthermore, prolonged passive stretching of the muscles might cause viscoelastic deformation and subsequent fatigue-like changes of the lumbar erector spinae muscles [50, 51], which is consistent with our observation of lower values of MDF in the presence of spinal tissue creep. Reduction in motor unit activation and average conduction velocity following passive stretching have indeed been described [52, 53]. Despite the overall tendency to observe creep effects in muscle activity distribution, a significant x-axis dispersion difference was only observed on the left side. In fact, x-axis dispersions are modest with higher levels of variability than y-axis dispersion, and the significant effect observed on the left side may be due to a slight misalignment of the arrays with respect to the fiber orientation and/or to changes in location of the innervation zone over time as previously suggested in other studies [54].

Dispersion values, however, were similar at the end of the fatigue task, whether there was spinal tissue creep or not. Dispersion in x-axis remained high after the deformation protocol, whereas dispersion in y-axis decreased in presence of spinal tissue creep. The physiological mechanisms underlying this phenomenon remain unclear. It could be hypothesized that the central nervous system, when muscle fatigue and spinal tissue creep are combined, strives to maintain vertebral stability using increased muscle activation, but less variable neuromuscular recruitment patterns.

### Recommendations for future studies

Low back muscle motor unit recruitment and adaptation under spinal tissue creep conditions should be investigated to document the physiological phenomenon underlying changes in muscle recruitment strategies. Additionally, since the effect of creep deformation on muscular stabilizing activity partially recovers up to 25% following a 10-minute rest [23] or up to 50% following a 25-minute rest [22], future studies should focus on documenting creep deformation effects during prolonged fatiguing exercises.

### Limitations

The study’s limitations include the participants’ status (young healthy adults), which may limit the generalization of results. Three participants were also excluded from EMG analyses due to high levels of EMG noise in recordings. Specifically, EMG signals from 2 participants could not be used as the ground electrode disconnected during the second Sorensen test. The other participant was not included in the analyses because EMG arrays failed to properly record EMG signals during the first Sorensen test.

ROM results should be interpreted with caution since differences between sitting and standing ROM assessments have been described. This could be explained by varying trunk muscle recruitment strategies between positions.

The effect of creep on RMS and MDF variables, and the interaction between fatigue and creep on the y-axis dispersion only reached statistical significance on one side of the trunk. This suggests that the study may have been underpowered. In this study, the presence of low back muscle fatigue was characterized by a marked decrease in the mean MDF slope. It is also known that MDF changes can be triggered by changes in intramuscular temperature [55]. Intramuscular temperature can also induce an increase in the spectral content of the sEMG signal. However, since a MDF decrease was observed, it is reasonable to suggest that acute fatigue effect superseded the temperature effect on MDF.

## Conclusions

Results from the current study indicate that combined muscle fatigue and creep deformation of spinal tissues lead to increases in muscle activity amplitude, as well as increases in muscle activity distribution. The impact of neuromuscular adaptations on spinal stability remains unclear. The current study provides information suggesting that prolonged deep trunk flexion can alter mechanical and neuromuscular functions of the lumbar components which may, over time, lead to the development or perpetuation of low back disorders.
